# Associations between Serum Aflatoxin-B1 and Anemia in Pregnant Women: Evidence from Guangxi Zhuang Birth Cohort in China

**DOI:** 10.3390/toxins13110806

**Published:** 2021-11-15

**Authors:** Lei Lei, Shun Liu, Ye Ye, Xiaoqiang Qiu, Dongping Huang, Dongxiang Pan, Jiehua Chen, Zhengmin Qian, Stephen Edward McMillin, Michael G. Vaughn, Xingxi Luo, Kaili Wu, Suyang Xiao, Jinxiu Li, Meiliang Liu, Yu Yang, Mingshuang Lai, Guanghui Dong, Xiaoyun Zeng

**Affiliations:** 1 Department of Epidemiology and Health Statistics, School of Public Health, Guangxi Medical University, Nanning 530021, China; gxmuleilei@163.com (L.L.); xqqiu9999@163.com (X.Q.); gxpandongxiang@163.com (D.P.); 14795591468@163.com (J.C.); gxmuluoxingxi@163.com (X.L.); kl202021003@163.com (K.W.); 15228110293@163.com (S.X.); gxmulijinxiu@163.com (J.L.); lk201720073@outlook.com (M.L.); yangyu_1997@163.com (Y.Y.); lms18777108295@163.com (M.L.); 2 Department of Maternal, Child and Adolescent Health, School of Public Health, Guangxi Medical University, Nanning 530021, China; liushun@gxmu.edu.cn; 3 Center for Disease Control and Prevention, Guangxi Liuzhou Iron & Steel Group Co., Ltd., Liuzhou 545002, China; gxyeye@163.com; 4 Department of Sanitary Chemistry, School of Public Health, Guangxi Medical University, Nanning 530021, China; dongpinghuang@gxmu.edu.cn; 5 Department of Epidemiology and Biostatistics, College for Public Health & Social Justice, Saint Louis University, St. Louis, MO 63103, USA; zhengmin.qian@slu.edu; 6 School of Social Work, College for Public Health & Social Justice, Saint Louis University, St. Louis, MO 63103, USA; stephen.mcmillin@slu.edu (S.E.M.); michael.vaughn@slu.edu (M.G.V.); 7 Department of Environmental and Occupational Health, School of Public Health, Sun Yat-sen University, Guangzhou 510080, China

**Keywords:** aflatoxin B1, hemoglobin, erythrocyte parameters, anemia, pregnant women, cohort study

## Abstract

Aflatoxin B1 (AFB1) is a common toxic mycotoxin and is detectable in pregnant women. Animal studies have revealed that AFB1 caused the lysis of erythrocytes and a decrease in hemoglobin. We conducted a prospective cohort study in Guangxi, China, in order to evaluate the association between AFB1 exposure and anemia in pregnant women during the entire pregnancy. A total of 616 pregnant women from the Guangxi Zhuang Birth Cohort were included in the study. Serum AFB1-albumin (AFB1-ALB) adduct levels were measured. The effect of AFB1-ALB adducts on hemoglobin (Hb), mean corpuscular volume (MCV), mean corpuscular hemoglobin (MCH), and mean corpuscular hemoglobin concentration (MCHC) were analyzed by using multivariable linear regression. The risks of anemia from AFB1-ALB adduct exposure were assessed by multivariable logistic regression. We found that the AFB1-ALB adduct was significantly associated with a decrease in Hb (β = −4.99, 95% *CI:* −8.42, −1.30), MCV (β = −4.58, 95% *CI:* −7.23, −1.94), MCH (β = −1.86, 95% *CI:* −2.87, −0.85), and MCHC (β = −5.23, 95% *CI:* −8.28, −2.17) in the first trimester with the third tertile of AFB1-ALB adducts when compared with the first tertile. Furthermore, the third tertile of the AFB1-ALB adduct significantly increased the risk of anemia by 2.90 times than compared to the first tertile in the first trimester (OR = 3.90, 95% *CI*: 1.67, 9.14). A significant positive does–response relationship existed between AFB1-ALB adduct levels and anemia risk (*P*_trend_ = 0.001). When dividing anemia types, we only found that the third tertile of AFB1-ALB adduct increased the risk of microcytic hypochromic anemia (MHA) in the first trimester (OR = 14.37, 95% *CI*: 3.08, 67.02) and second trimester (OR = 4.75, 95% *CI*: 1.96, 11.51). These findings demonstrate the correlation between maternal AFB1 exposure during early pregnancy and risk of anemia, especially MHA, and during different trimesters in Southern China. More efforts should be made to diminish AFB1 exposure for pregnant women.

## 1. Introduction

Aflatoxins are a harmful mycotoxin [1]. Among the various types of aflatoxins, aflatoxin B1(AFB1) is the predominant form with the most toxic effect [2]. AFB1 is produced by Aspergillus, which usually contaminates a wide range of agricultural products, such as grain, nuts, or spices, especially in tropical and subtropical climatic regions [3]. AFB1 is not only known as one of pathogenic factors for liver cancer, but it is also associated with other chronically adverse effects [4], including immunity suppression, nutrient absorption, and metabolism disturbance.

Aflatoxin contamination has been a concern in China due to the high consumption of cereal products in daily food and peanut as the major oil crop. Indeed, a recent study reported that the contamination of peanut oil with AFB1 is relatively high in provinces experiencing a subtropical climate [5]. Fan et al. [6] assessed the mycotoxins exposure of the population in Nanjing, China, and noted that AFB1 was common in both human serum and urine. As such, AFB1 contaminations are posing a threat to public health in the Chinese population.

Notably, AFB1 exposure is ubiquitous in the natural environment throughout the world, and disconcertingly it has been detectable in pregnant women [7,8,9]. Accumulating evidence indicates a link between prenatal AFB1 exposure and adverse pregnancy and birth outcomes, including maternal anemia and intrauterine growth restriction [10,11]. Anemia is a widespread health problem, affecting 27% of the population worldwide and 22% of pregnant women in China [12]. In turn, anemia is confirmed to be connected with fetal growth restriction [13], premature birth, and low birth weight [14]. Animal studies have revealed that AFB1 has impaired the morphology of erythrocytes by inducing oxidant stress and caused a decrease in hemoglobin concentration [15,16]. However, the effect of AFB1 has not been extensively studied in humans. Only one cross-sectional research study reported the relationship between AFB1 exposure and anemia among pregnant woman in Ghana, Africa [17]. This study only examined the association between AFB1 exposure and anemia during the delivery stage. While informative, it is of critical importance to study the associations of AFB1 exposure across the entire pregnancy. To the best of our knowledge, no studies have ever investigated the association of AFB1 exposure with anemia across pregnancy trimesters.

The site in the present study is Guangxi, a province in Southern China that is a subtropical climate region and is composed of a large Zhuang ethnic population. Considering the high prevalence and potential health risk of AFB1 exposure, we hypothesized that AFB1 exposure is associated with maternal anemia. We examined the association between AFB1 exposure and anemia, as well as different types of anemia, across the three trimesters of pregnancy in an ongoing prospective birth cohort study.

## 2. Methods

### 2.1. Study Population

**Figure 1:**
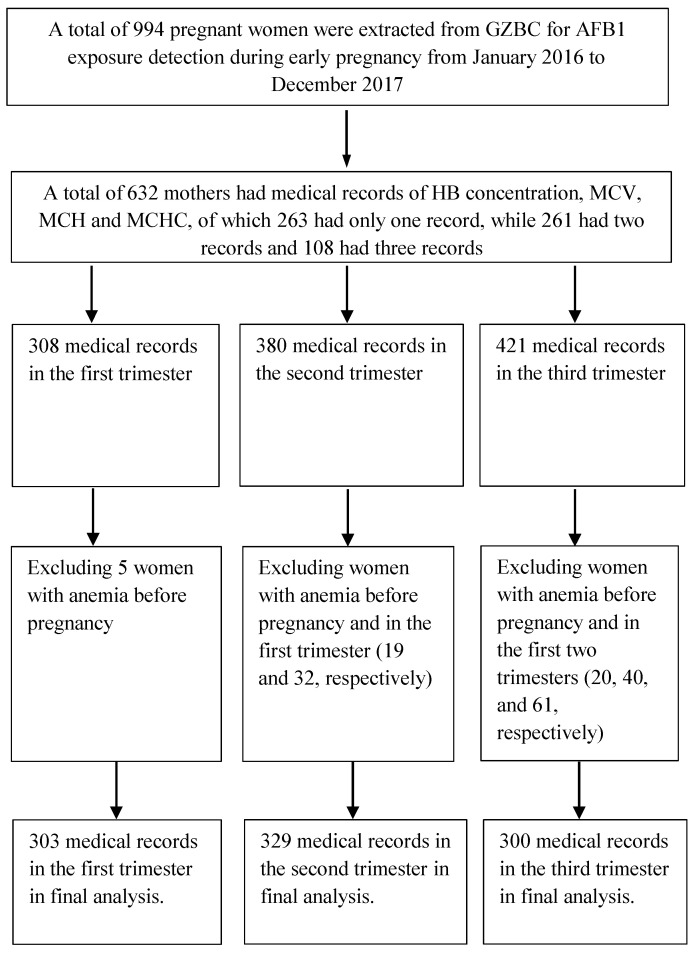
Flowchart of the study population.

This study was part of the Guangxi Zhuang Birth Cohort (GZBC), an ongoing prospective birth cohort study of the Zhuang population in Guangxi province, China. The details of this cohort have been described previously [18,19]. In the present study, we extracted cohort data for 994 pregnant women with live singletons from January 2016 to December 2017 for AFB1 exposure in early pregnancy. Of the 994 women, 632 mothers had medical records of hemoglobin (Hb) concentrations and erythrocyte parameters (308, 380, and 421 records in the first, second, and third trimesters, respectively). Among them, 263 mothers had records in one trimester, while 369 mothers had overlapping records during trimesters (261 mothers had records in two trimesters, and 108 mothers had records in three trimesters). For the final analysis, 303 records were retained in the first trimester after excluding 5 women with anemia before pregnancy; 329 were retained in the second trimester after excluding those with anemia before pregnancy and in the first trimester (19 and 32, respectively); and 300 were retained in the third trimester after excluding those with anemia before pregnancy and in the first two trimesters (20, 40, and 61, respectively) (Figure 1). In the final analysis, we included 616 mothers. Of the 616 mothers, 351 mothers had records in one trimester, while 265 mothers had overlapping records during trimesters (214 mothers had records in two trimesters, and 51 mothers had records in all three trimesters).

This study has been approved by the ethical committee of Guangxi medical university (No.20140305-001). All the participants signed informed consents and agreed to donate their blood samples at enrollment.

### 2.2. Data Collection

Baseline information including demographic characteristics (i.e., maternal age, occupation, pre-pregnancy weight, and height), lifestyle (i.e., regular physical activity in early pregnancy, pre-pregnancy alcohol consumption, and smoking), and pre-pregnancy folic acid supplement use were obtained by using a standardized questionnaire conducted by trained investigators at enrollment. Pre-pregnancy BMI was calculated by using self-reported height and pre-pregnancy weight. Maternal information (i.e., gravidity, parity, and pregnancy complications) was collected from medical records. Gestational age (weeks) was determined according to the last menstrual cycle. Ultrasound was used to estimate gestational age if the last menstrual cycle was not certain. Infant information (i.e., gender, birth weight, birth height, and gestational weeks at delivery) was collected from Guangxi maternal and child healthcare system.

Hb concentrations and erythrocyte parameters, including mean corpuscular volume (MCV), mean corpuscular hemoglobin (MCH), and mean corpuscular hemoglobin concentration (MCHC), were obtained from medical records. When there was more than one record in the same trimester (9, 16, and 25 mothers in the first, second, and third trimesters, respectively), we used the mean values as the precise levels.

### 2.3. AFB1 Exposure Measurements

All participants provided a blood sample at enrollment. The blood samples were centrifuged, separated, and then stored at −80 °C until further analyses. We used a double- antibody-sandwich-enzyme-linked immunosorbent assay (ELISA) to quantify the serum AFB1-ALB adduct. AFB1-ALB adduct is an effective biomarker for assessing human AFB1 exposure [20,21,22,23]. It can reflect chronic AFB1 exposure around 1–3 months due to its long half-life [24] and is stable during deep-frozen storage. The ELISA method is a routine and sensitive screening tool for monitoring AFB1-ALB adduct and has been previously described [25]. We purchased the AFB1-albumin ELISA kit from MEIMAIN (Nanjing, China) and performed testing strictly according to the manufacturer’s instructions. An enzyme-plate analyzer was used to determine the OD value at 450 nm. The concentration of AFB1-ALB adduct in the samples was then calculated by the standard curve.

### 2.4. Statistical Analysis

Demographic characteristics were described using mean (±SD) for continuous variables and frequency (*n* or %) for categorical variables. A multivariable linear regression model was conducted to explore the association between AFB1-ALB adduct and levels of Hb, MCV, MCH, and MCHC in different trimesters. A multivariable logistic regression model was conducted to evaluate the association between AFB1-ALB adduct levels and the risk of anemia in different trimesters. Serum AFB1-ALB adduct concentration was modeled as a categorical variable (tertiles), and the lowest tertile was set as the reference. Trend tests were also used to explore a dose–response relationship.

For multivariable linear regression and multivariable logistic regression model, we included covariates identified to be associated with anemia or serum AFB1-ALB adduct in a previous study [26], including maternal age (<22, 22–35, and >35 years); pre-pregnancy BMI (underweight: <18.5; normal: 18.5–23.9; overweight: ≥24 kg/m^2^); pre-pregnancy folic acid supplement use (yes; no); gravidity(primigravida and multigravida); parity (0, ≥1); regular physical activity in early pregnancy (yes; no); alcohol consumption in early pregnancy (yes; no); passive smoking in early pregnancy (yes; no); gestational age at blood test for Hb, MCV, MCHC, and MCHC (continuous); and infant gender (boy; girl) (except in model stratified by infant gender). In addition, we adjusted sampling seasons (spring, summer, autumn, and winter) because seasonal related factors including different temperatures can affect levels of AFB1 [3].

In order to determine the robustness of the main analyses, we excluded participants with abnormal liver function, diabetes, hypertension, and preeclampsia (38, 40, and 47 mothers in the first, second, and third trimesters, respectively) in sensitivity analyses. All analyses were conducted using SPSS 22.0. A two-sided *p* < 0.05 was considered statistically significant.

## 3. Results

### 3.1. Characteristics of the Included Participants

Most of the participants (93.8%) were Zhuang ethnicity, and 79.5% were multigravida. 5.5% percent of them drank alcohol, and 46.4% had exposure to second-hand smoking during early pregnancy; 12.8% took folic acid before pregnancy, and 41.4% reported regular physical exercise during early pregnancy. The mean ± SD maternal age (years) and pre-pregnancy BMI (kg/m^2^) were 28.80 ± 5.66 and 20.84 ± 2.53, respectively. Among infants, there were 330 boys and 286 girls. Mean gestational age at birth was 38.67 ± 1.60 weeks. The mean (±SD) birth weight and birth length were 3089.89 ± 428.39 g and 49.94 ± 1.92 cm (cm). There were 34 low birth weight (LBW) infants; 15.3% of the infants were small for gestational age (SGA), while 7% were large for gestational age (LGA) (Table 1).

### 3.2. Serum AFB1-ALB Adduct Levels, Hb, MCV, MCH, and MCHC Concentrations and Anemia Prevalence

The detection rate of serum AFB1-ALB adduct was 100%. Mean (±SD) serum AFB1-ALB adduct concentration (pg/mL) was 557.39 ± 105.07. There was no significant difference between the study population and the excluded group in serum AFB1-ALB adduct concentrations. Mean Hb concentrations and erythrocyte parameters in different trimesters are shown in Table 2. Anemia was defined as hemoglobin <110 g/L. Based on this criterion, 19%, 34.3%, and 28.7% of the pregnant women had anemia during the first, second, and third trimester, respectively.

### 3.3. Associations between AFB1 Exposure and Hb, MCV, MCH, and MCHC Levels

**Figure 2:**
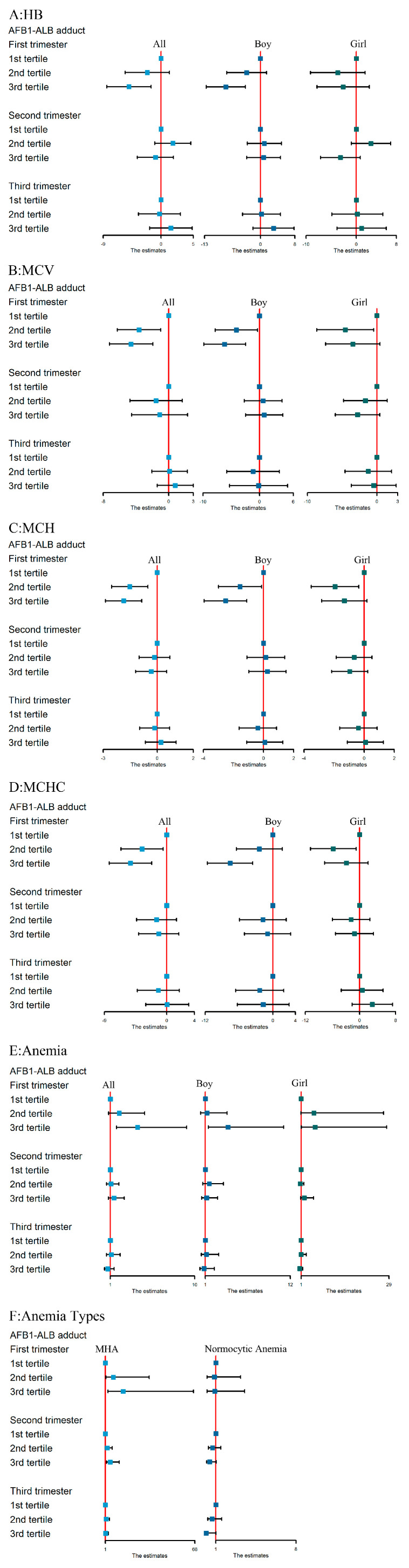
Associations of maternal serum levels of AFB1-ALB adduct with concentrations of Hb (**A**), MCV (**B**), MCH (**C**), MCHC (**D**), anemia (**E**), and anemia types (**F**) in fully adjusted models. Note: 1st tertile: ≤491.68 pg/mL; 2nd tertile: 491.68–603.41 pg/mL; 3rd tertile: ≥603.41 pg/mL. Serum levels of AFB1-ALB adduct were treated as independent variable, while Hb, MCV, MCH, and MCHC were treated as dependent variables in multivariable linear regression model. Serum levels of AFB1-ALB adduct were treated as independent variable, while anemia and anemia types were treated as dependent variables in multivariable logistic regression model. Abbreviations: Hb, hemoglobin; MCV, mean corpuscular volume; MCH, mean corpuscular hemoglobin, MCHC, mean corpuscular hemoglobin concentration; MHA, microcytic hypochromic anemia. Adjusted factors included pre-pregnancy BMI; maternal age; folic acid supplement pre-pregnancy; gravidity; parity; regular physical activity in early pregnancy; alcohol consumption and passive smoking in early pregnancy; sampling season; gestational age at blood test for Hb, MCV, MCHC, and MCHC; and infant gender (except in model stratified by infant gender).

Multiple linear regression analyses of the association among AFB1-ALB adduct and concentrations of Hb, MCV, MCH, and MCHC in the three trimesters are shown in Figure 2. In the first trimester, we observed a significant dose-dependent, inverse relationship between the AFB1-ALB adduct level and Hb, MCV, MCH, and MCHC in the entire population in the fully adjusted model. Pregnant women within the third tertile of AFB1-ALB adduct had a significant decrease in Hb (β = −4.99, 95% *CI:* −8.42, −1.30), MCV (β = −4.58, 95% *CI:* −7.23, −1.94), MCH (β = −1.86, 95% *CI:* −2.87, −0.85), and MCHC (β = −5.23, 95% *CI:* −8.28, −2.17) than those with the first tertile in the first trimester. We also observed a dose–response relationship between AFB1-ALB adduct and levels of Hb, MCV, MCH, and MCHC (the tests of *P*_trend_ were 0.005, 0.001, <0.001, and 0.001, respectively). Stratified by infant gender, we only observed a significant association between AFB1-ALB adduct and these outcomes among boys. No significant associations were observed in the second and third trimesters.

### 3.4. The Association between AFB1 Exposure and Maternal Anemia Risk

Figure 2 presents the association between AFB1-ALB adduct exposure and anemia during the three trimesters. Consistently, when compared to the first tertile of AFB1-ALB adduct, mothers in the third tertile had a significant increasing risk of anemia by 2.90 times in the first trimester (OR = 3.90, 95% *CI*: 1.67, 9.14). Furthermore, trend tests showed that anemia risk was significantly increased by the level of AFB1-ALB concentrations (*P*_trend_ = 0.001). After stratification by infant gender, we also observed significant associations in mothers with boy infants in the first trimester. However, this relationship disappeared in the second and third trimesters.

### 3.5. The Association between AFB1 Exposure and Risk of Different Anemia Types

According to the levels of MCV, MCH, and MCHC, we classified anemia into the following three types: macrocytic anemia, normocytic anemia, and macrocytic hypochromic anemia (MHA). As the proportion of microcytic anemia was low in our study population (1.0%, 4.90%, and 0 in the first, second, and third trimesters, respectively), we only analyzed the association between AFB1-ALB adduct and normocytic anemia and MHA. When compared to those in the first tertile of AFB1-ALB adduct, mothers in the third tertile had a significant increasing risk of MHA by 13.37 times in the first trimester (OR = 14.37, 95% *CI*: 3.08, 67.02) and 3.75 times in the second trimester (OR = 4.75, 95% *CI*: 1.96, 11.51). Both the trend tests showed a dose–response relationship (both *P*_trend_ < 0.001) (Figure 2). However, the relationship in the third trimester disappeared. No significant association was found between AFB1-ALB adduct and normocytic anemia in the three trimesters.

**Figure 3:**
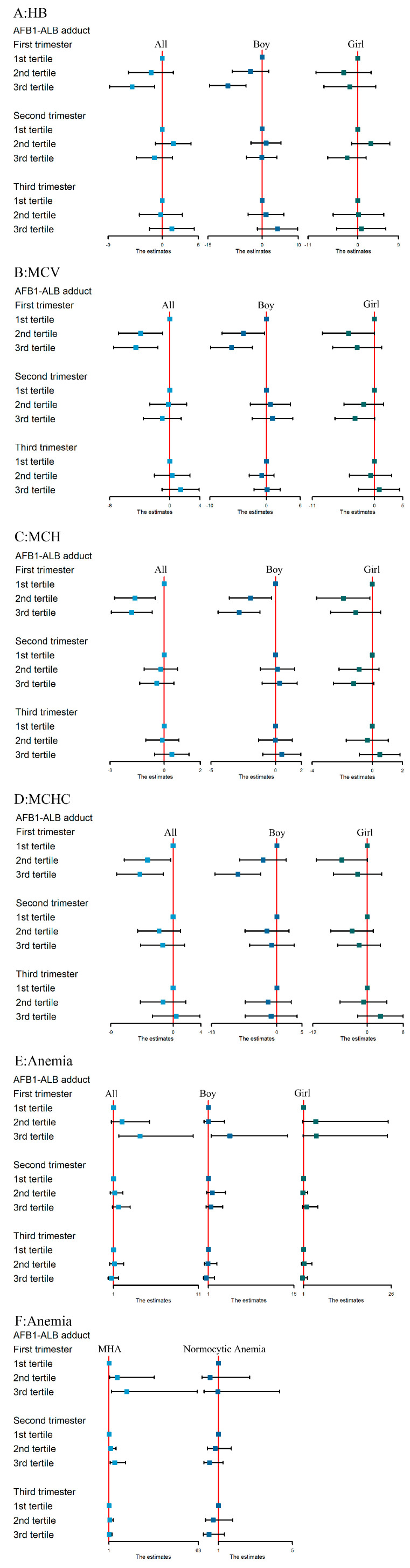
Associations of maternal serum levels of AFB1-ALB adduct with concentrations of Hb (**A**), MCV (**B**), MCH (**C**), MCHC (**D**), anemia (**E**), and anemia types (**F**) in fully adjusted models when excluding participants with abnormal liver function, diabetes, hypertension, and preeclampsia. Note: 1st tertile: ≤491.68 pg/mL; 2nd tertile: 491.68–603.41 pg/mL; 3rd tertile: ≥603.41 pg/mL. Serum levels of AFB1-ALB adduct were treated as independent variable, while Hb, MCV, MCH, and MCHC were treated as dependent variables in multivariable linear regression model. Serum levels of AFB1-ALB adduct were treated as independent variable, while anemia and anemia types were treated as dependent variables in multivariable logistic regression model. Abbreviations: Hb, hemoglobin; MCV, mean corpuscular volume; MCH, mean corpuscular hemoglobin, MCHC, mean corpuscular hemoglobin concentration; MHA, microcytic hypochromic anemia. Adjusted factors included pre-pregnancy BMI; maternal age; folic acid supplement pre-pregnancy; gravidity; parity; regular physical activity in early pregnancy; alcohol consumption and passive smoking in early pregnancy; sampling season; gestational age at blood test for Hb, MCV, MCHC and MCHC; and infant gender (except in model stratified by infant gender).

When we excluded participants with abnormal liver function, diabetes, hypertension, and preeclampsia (Figure 3), the results were consistent with the main findings.

## 4. Discussion

Mounting evidence suggests that AFB1 exposure is associated with a wide array of adverse pregnancy and birth outcomes, including maternal anemia and intrauterine growth restriction. We surmounted previous limitations in the literature and investigated the association of AFB1 exposure during pregnancy with maternal anemia in different trimesters using a prospective cohort. We found that elevated serum AFB1-ALB adduct was associated with decreased Hb, MCV, MCH, and MCHC and increased risk of anemia, especially microcytic hypochromic anemia, in pregnant women. Our study provides new evidence that AFB1 exposure may be a risk factor for anemia during pregnancy.

In the current study, AFB1-ALB adducts were detected in the serum of all sampled mothers in early pregnancy, indicating a potential health risk of aflatoxin exposure during pregnancy in Guangxi. The levels of AFB1-ALB adduct in this study were lower than that in children aged 6–9 years in the West Kiang region of Gambia (45.38 pg/mg) [27]. However, it was higher than that of rural residents in Huai’an, Jiangsu province, China (44.48 pg/mL) [28]. These differences may be due to the differences in study population, regional climate [3], socio-economic status, dietary habits, and food storage practices [29].

Only one cross-sectional study has reported the association between AFB1-ALB adduct and anemia among pregnant women [17]. In the present study, we not only observed a similar association but also observed the associations of AFB1-ALB adduct with an increased risk of microcytic hypochromic anemia, which was characterized as low MCV, MCH, and MCHC. Our findings are consistent with a previous animal study in which rabbits fed with aflatoxin had reductions in Hb, MCV, MCH, and MCHC [30]. Additionally, our results suggest that the first and second trimesters are periods of heightened vulnerability to aflatoxin exposure. Since we collected blood samples only at a single time-point and aflatoxins may transfer to the placenta during pregnancy [9,31], longitudinal observations of aflatoxin exposure during the entire pregnancy are needed in future studies. Furthermore, we observed a sex-specific effect that mothers with male infants were more likely to be affected by aflatoxin. This is congruent with previous reports finding that the metabolism of aflatoxin was different between sexes, and males were more susceptible to aflatoxin [32,33]. However, the underlying mechanisms for this difference is still unclear.

Erythrocytes lysis and iron deficiency have been found to be the main causes of microcytic hypochromic anemia [34]. In animals, AFB1 was found to cause the lysis of erythrocytes [35] and disturb iron absorption [36]. Furthermore, accumulating evidence has suggested that oxidant stress [15] and disruption of immune homeostasis [37] may be the mechanism by which AFB1 is involved in the above processes. It has been reported that AFB1 increased oxidant level [38] and decreased antioxidants in extrahepatic tissue and cells (i.e., kidney, erythrocytes, kidney, heart, brain, and spleen) in rats [39], resulting in the impairment of morphology and membrane of erythrocytes, which causes the hemolysis of erythrocytes and then anemia. In addition, AFB1 has long been known as an immunosuppressant [40,41], and the immunostimulatory effects of AFB1 have also been reported in a recent study [37]. AFB1 exposure decreases serum anti-inflammatory cytokines (IL-4 and IL-10) and upregulates inflammatory cytokine (IL-6 and TNF-α) in rats [42,43,44]. IL-6 further stimulates the synthesis of hepcidin [45] by blocking iron absorption and macrophage iron recycling, resulting in iron deficiency and, in turn, anemia [46,47]. The above evidence reveals a strong convergent association between AFB1 exposure and microcytic hypochromic anemia. What is of critical importance, however, is that these findings need to be further confirmed in studies with human populations and cells.

The present study findings should be interpreted in the context of several limitations. First, we were only able to measure AFB1-ALB adducts from a single blood sample collected from the first trimester of pregnancy, and this may not be able to reflect AFB1 exposure during the entire pregnancy very well. However, we recruited permanent residents at enrollment with the consideration that the participants were under a relatively stable climate and diet; AFB1 exposure may be also under a relative level. Measuring AFB1-albumin adducts during all three trimesters can provide an overall picture of AFB1 exposure during the entire pregnancy and would better illustrate the effect of aflatoxins on anemia. Second, measurement errors are possible because we collected medical records of Hb and erythrocyte parameters from different hospitals. However, all the tests of Hb and erythrocyte parameters were performed by trained hospital staff using a hemocyte analyzer strictly according to the manufacturer’s operation procedures and under rigorous quality control. Lastly, we did not test the serum concentrations of iron and diagnose the hemolysis; thus, we cannot identify the underlying mechanisms.

Our prospective study found widespread exposure to AFB1 among pregnant women in Guangxi, China. We observed that AFB1 exposure was significantly associated with decreased levels of Hb and erythrocyte parameters in the first trimester of pregnancy, as well as an increased risk of microcytic hypochromic anemia during the first and second trimesters of pregnancy in the same population.

Considering the adverse effect on pregnancy [11] and birth outcome [48], more efforts should be taken to reduce the exposure to aflatoxins of pregnant women. Multiple public health intervention should be applied to reduce the level of aflatoxins in key crops, such as maize and peanut [49]. Agricultural interventions can be applied either in the field (preharvest) or in storage and transportation (postharvest) [50] in order to lower foodborne exposure. On the other hand, food diversity will lower the risks of exposure by reducing the intake of commonly contaminated food [49]. We suggest that pregnant women consume food with low risk of aflatoxins contamination, both at periods of childbearing and breastfeeding.

## Figures and Tables

**Table 1:** The characteristics of the participants (*n* = 616).

Characteristic	Mean ± SD or *n* (%)
Mothers	
Pre-pregnancy BMI (kg/m^2^)	20.84 ± 2.53
Ethnic	
Zhuang	578 (93.8)
Han	38 (6.2)
Gravidity	
Primigravida	126 (20.5)
Multigravida	490 (79.5)
Parity	
0	227 (36.9)
≥1	389 (63.1)
Pre-pregnancy folic acid supplement	
No	537 (87.2)
Yes	79 (12.8)
Alcohol consumption pre-pregnancy	
No	582 (94.5)
Yes	34 (5.5)
Passive smoking in early pregnancy	
No	330 (53.6)
Yes	286 (46.4)
Regular physical activity	
No	361 (58.6)
Yes	255 (41.4)
Maternal age (years)	28.80 ± 5.66
Infants	
Infant gender	
Boy	330 (53.6)
Girl	286 (46.4)
Gestational age at birth (weeks)	38.67 ± 1.60
Birth weight (g)	3089.89 ± 428.39
Birth length (cm)	49.94 ± 1.92
LBW	34 (5.5)
SGA	94 (15.3)
LGA	43 (7.0)

Abbreviations: SD, standard deviation; BMI, body mass index; LBW, low birth weight; SGA, small for gestational age; LGA, large for gestational age.

**Table 2:** The distribution of Hb concentrations and erythrocyte parameters among the three trimesters.

Variables	First Trimester (*n* = 303)	Second Trimester (*n* = 329)	Third Trimester (*n* = 300)
Hb (g/L)	118.86 ± 12.36	113.37 ± 10.58	115.48 ± 11.97
MCV (fl)	85.48 ± 9.45	88.12 ± 8.66	87.29 ± 7.80
MCH (pg)	28.11 ± 3.64	28.69 ± 3.21	27.98 ± 3.06
MCHC (g/L)	328.51 ± 10.96	324.17 ± 11.03	319.95 ± 11.44
Anemia (*n*%)	58 (19.1)	113 (34.3)	86 (28.7)

Abbreviations: Hb, hemoglobin; MCV, mean corpuscular volume; MCH, mean corpuscular hemoglobin; MCHC, mean corpuscular hemoglobin concentration.
